# Targeting PI3K, FGFR, CDK4/6 Signaling Pathways Together With Cytostatics and Radiotherapy in Two Medulloblastoma Cell Lines

**DOI:** 10.3389/fonc.2021.748657

**Published:** 2021-09-24

**Authors:** Monika Lukoseviciute, Henrietta Maier, Eleni Poulou-Sidiropoulou, Erika Rosendahl, Stefan Holzhauser, Tina Dalianis, Ourania N. Kostopoulou

**Affiliations:** Department of Oncology-Pathology, Karolinska Institutet, Stockholm, Sweden

**Keywords:** childhood cancer, medulloblastoma (MB), targeted therapy, PI3K inhibitors, FGFR inhibitors, CDK4/6 inhibitors

## Abstract

**Objectives:**

Medulloblastoma (MB) is treated with surgery and chemotherapy, with or without irradiation, but unfortunately >20% of the patients are not cured, and treatment comes with serious long-term side effects, so novel treatments are urgently needed. Phosphoinositide 3-kinases (PI3K), fibroblast growth factor receptors (FGFR), and cyclin-D kinases (CDK) play critical roles in cancer, and especially PI3K is crucial in MB, so here targeted therapies against them were explored.

**Methods:**

MB cell lines DAOY and UW228-3 were exposed to PI3K (BYL719), FGFR (JNJ-42756493), and CDK4/6 (PD-0332991) inhibitors, as single or combined treatments, and their viability, cell confluence, apoptosis, and cytotoxicity were examined. Moreover, the inhibitors were combined with cisplatin, vincristine, or irradiation.

**Results:**

Single treatments with FGFR, PI3K, or CDK4/6 inhibitors decreased viability and proliferation slightly; however, when combining two inhibitors, or the inhibitors with irradiation, sensitivity was enhanced and lower doses could be used. A more complex pattern was obtained when combining the inhibitors with cisplatin and vincristine.

**Conclusions:**

The data suggest that combination treatments with PI3K, FGFR, and CDK4/6 inhibitors for MB could be beneficial and their use should be pursued further. Likewise, their combination with irradiation gave positive effects, while the addition of cisplatin and vincristine resulted in more complex patterns, which need to be investigated further.

## Introduction

Yearly, around 300,000 children aged 0 to 19 years old are diagnosed with cancer, and notably brain and central nervous system (CNS) tumors are among the most frequent childhood solid tumors (1, 2). Medulloblastoma (MB), the subject of the present report, usually occurs in the cerebellum, and accounts for 16–25% of all CNS tumors in children (3–6). Based on molecular advances in genomics, gene expression profiles, and DNA methylation analysis, today MBs are separated into four major categories: Wing-less/Integrated (WNT), Sonic Hedgehog (SHH), Group 3 and Group 4 (7–9). Group 4 and SHH-activated MB, with intermediate prognosis, dominate, and include ~35 and 30%, respectively, of all MB. Group 3 tumors, with the worst prognosis, account for 25% cases, while WNT, with the best prognosis, encompasses 10% of all MBs (7, 10, 11).

Treatment of MB consists of surgery, chemotherapy (CT), and usually radiation therapy (RT), depending also on the age of the individual; however, despite all efforts, around 30% of the patients succumb to disease, and those who survive often suffer from severe long-term side effects (12, 13). The latter primarily include neurological deficits and endocrine disorders, but also secondary cancers can arise (13). To improve survival and reduce side effects, novel combination therapies are needed, and this study is part of this pursuit.

Lately, great progress has been obtained in cancer treatment, and much of the advances have been due to the use of immunotherapy with PD1/PDL1 inhibitors, but also targeted therapy has been helpful with most experience in adult cancer (14, 15). For example, phosphatidylinositol 3-kinase (PI3K) and Cyclin D kinase 4/6 (CDK4/6) inhibitors have been used against metastatic breast cancer [with PI3K catalytic subunit alpha (PIK3CA) mutations], and fibroblast growth factor receptor (FGFR) inhibitors have been used against bladder cancer (with FGFR3 mutations) (16–19). Moreover, PI3K inhibitors have e.g. been combined with CDK4/6 inhibitors in metastatic breast cancer (20).

The PI3K pathway has been investigated to some extent in childhood cancer, while this is not as much the case for CDK4/6, and even less so for members of the FGFR family (21, 22). Mutations and gene amplifications of the PIK3CA gene have been documented in many adult tumors, as well as in MB (23, 24). Clearly, since PI3K inhibitors have been used alone, or combined with CT in adult cancer, they could potentially also be beneficial for MB treatment (13, 25–27). Moreover, the use of CK4/6 alone, or together with PI3K inhibitors, or CT, as well as the effects of FGFR inhibitors may also be of interest to explore more extensively for possible treatment of recurrent MB. In fact, one clinical trial investigates the use of the CDK4/6 inhibitor ribociclib together with the cytostatic gemcitabine, or together with the small molecule trametinib, or with the Hh antagonist sonidegib, respectively, to treat recurrent or progressive MB (NCT03434262). Other trials combine the CDK4/6 inhibitor palbociclib, ribociclib, or abemaciclib, respectively, together with the alkylating agent temozolomide and the cytotoxic alkaloid irinotecan (NCT03709680, NCT04238819).

Based on the above progress, we recently examined the effects of PI3K and FGFR inhibitors in childhood neuroblastoma (NB) and MB cell lines (with/without PI3K mutations) *in vitro* and demonstrated that these inhibitors and especially in combination efficiently decreased viability as well as inhibition in the increase in cell confluence of the cell lines (28–30). The obtained data suggested that combinations of PI3K and FGFR inhibitors could be of potential use for MB and NB, even without the corresponding mutations. Notably, in the first two of these three studies, thereby including the one on MB-cell lines, the inhibitors that were used were not certified by the Food and Drug Agency (FDA) (28, 29). However, since then, the FDA has approved the PI3K inhibitor BYL719 and the FGFR inhibitor JNJ-42756493 for use in breast cancer and urinary bladder cancer (17, 19).

Consequently, it is of great importance to accumulate more knowledge on the potential sensitivity of MB to FDA-approved PI3K, CDK4/6, and FGFR inhibitors, alone or combined, or in different combinations together with clinically used cytostatic drugs cisplatin or vincristine, or RT. Here, we therefore tested two frequently used MB cell lines, DAOY and UW228-3, the former with, and the latter without, a PI3K mutation, but both with TP53 mutations, and thereby representative for aggressive MB, for their sensitivity to inhibitors of PI3K (BYL719), FGFR (JNJ-42756493), or CDK4/6 (PD-0332991) alone, or in combination, and together with cisplatin, vincristine, or RT.

## Materials and Methods

### Tumor Cell Lines and Culture Conditions

MB group SHH cell lines, DAOY, purchased from ATCC, and UW228-3, kindly provided by Prof. M. Nistér (Karolinska Institutet), were used in this study. According to gene bank https://depmap.org/portal/, neither of the cell lines had any FGFR3 mutations or MYC amplification, while DAOY had an in-frame deletion with a non-conserving PIK3R1 mutation, and they have both a mutation in TP53. DAOY, cultured in Minimum Essential Media (MEM), and UW228-3 in Dulbecco**’**s Modified Eagle Medium (DMEM/F-12), with both media supplemented with 10% fetal bovine serum (FBS), 1% L-glutamine, 100 U/ml penicillin, and 100 µg/ml streptomycin, were maintained at 37°C in a humidified incubator with 5% CO_2_. Media and FBS were purchased from Gibco, Waltham, MA, USA.

### Cell Seeding and Treatment

DAOY and UW228-3 were seeded at 2.5 × 10^3^ and 5 × 10^3^ cells, respectively, in 90–200 µl medium/well for the different assays, and the edges were filled with medium to avoid edge effects. Penicillin and streptomycin were excluded from the media in the different assays to avoid any interference with the drugs.

### Inhibitors

Alpelisib (BYL719) used as PI3K inhibitor, JNJ-42756493 as FGFR inhibitor, and PD-032991 as CDK4/6 inhibitor were purchased from Selleckhem Chemicals, Munich, Germany, and introduced 24 h after cell seeding. The concentrations of the drugs were as follows: for BYL719, 1.0–10 µM; JNJ-42756493, 1.0–10.0 μM; and PD-032991, 5.0–20 μM.

### Cytotoxic Agents

Stocks of cisplatin (Accord Healthcare Limited, Middlesex, UK) and vincristine (Oncovin, Pfizer, USA) were diluted in PBS, and the used concentrations were 1–10 μM for cisplatin and 5 nM–1 μM for vincristine.

### Irradiation

Cells at 2.5×10^3^ (DAOY) and 5×10^3^ (UW228-3) were treated 24 h after seeding, and then they were exposed to 0, 2, or 10 Gy 3 h after treatment with the X-Rad 225XL machine (PXi Precision X-ray, North Brandford, USA).

### WST-1 Viability Assay

Cell viability was followed by the WST-1 assay (Roche Diagnostics, Mannheim, Germany). Here, WST-1 (tetrazolium salt) is cleaved to formazan by cellular enzymes in viable cells as presented before in detail (29).

### Assessing Cell Confluence, Cell Cytotoxicity, and Apoptosis Assays

#### Assessing Cell Confluence

Cells seeded in 200 μl medium/well in a 96-well plate, were placed into the IncuCyte S3 Live−Cell Analysis System (Essen Bioscience, Welwyn Garden City, UK) for up to 72 h after seeding. The machine was set to scan the plates and take pictures every 2 h, with PBS as control and culture medium as background. Cell confluence was followed in these images.

#### Cell Cytotoxicity and Apoptosis Assays

IncuCyte Red Cytotoxicity reagent and IncuCyte Caspase-3/7 Green Apoptosis assay (both from Essen Bioscience, Welwyn Garden City, UK) were utilized to follow cytotoxicity and apoptosis. After 24 h of seeding, the medium was replaced with fresh medium containing the cytotoxicity reagent (final concentration of 250 nM per well) and the apoptosis reagent at a ratio of 1:1,000. The drugs were added thereafter, and for more details see (29, 30).

### Statistical Analysis

To estimate the effects of the combinational treatments, the effect-based approach **“**Highest Single Agent**”** and dose-effect-based approach **“**median-effect method**”** (based on Loewe Additivity) were used (31, 32). A combination index (CI) CI of **<**1 was considered as a positive and a CI of **>**1 as a negative combinational effect. Additionally, the possible additive, synergistic, or antagonistic effects of the combined treatments were analyzed using the median-effect method of Chou (Chou-Talalay method) as described before (29). Here the CI<0.70 defined as synergy, CI<1.45 as antagonism, and values in between as additive effects, according to the recommendations of the ComboSyn software. To determine the effects of single or combination treatments, a multiple t-test accompanied by a correction for multiple comparison of the means conferring to the Holm-Sidak method was performed as described in detail previously (29).

## Results

### Viability After Single and Combined Treatments With PI3K, FGFR, and CDK4/6 Inhibitors BYL719 (Alpelisib), JNJ-42756493 (Erdafitinib), and PD-0332991 (Palbociclib), Respectively, of MB Cell Lines DAOY and UW228-3

**Figure 1:**
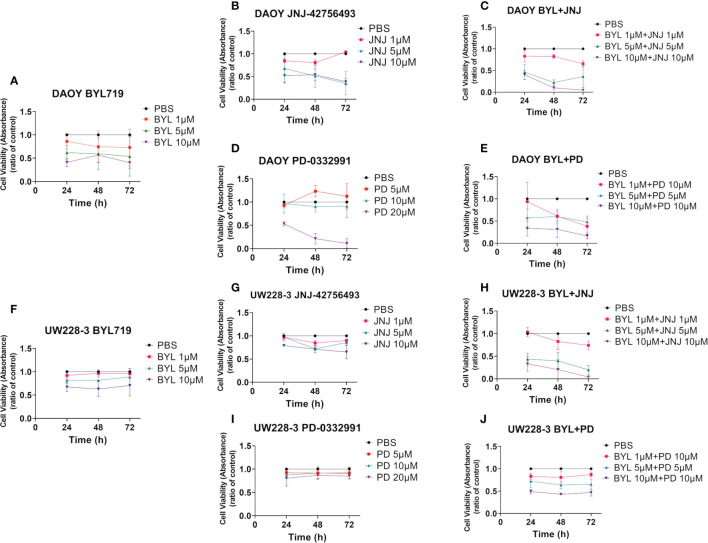
WST-1 viability assays after single and combined treatment of PI3K, FGFR, and CDK4/6 inhibitors (BYL719, JNJ-42756493, PD-033299, respectively) on two MB cell lines (DAOY and UW228-3). Viability analysis of DAOY and UW228-3 respectively measured as absorbance, after single treatments for 24, 48, and 72 h with BYL719 **(A, F)**, FGFR JNJ-42756493 **(B, G)**, and PD-0332991 **(D, I)**, respectively. Combination treatments are shown in **(C, E)** for DAOY and **(H, J)** for UW228-3. The graphs represent mean values ± standard deviation (SD) from three experimental runs per cell line. BYL, BYL719; JNJ, JNJ-42756493; PD, PD-0332991.

Data from at least three experiments with WST-1 viability assays following viability of DAOY and UW228-3 after single treatments with BYL719 (1–10 µM), JNJ-42756493 (1–10 μM), or PD-0332991 (5–20 μM), for 24, 48, and 72 h are compiled in **Figure 1**. In addition, corresponding data of combination treatments with BYL719, and JNJ-42756493 (both 1–10 µM) and PD-0331991 (5–10 µM) are also depicted in **Figure 1**, while combinations of JNJ-42756493 (5–10 µM) and PD-0331991 (1–10 µM) are shown in **Supplementary Figure 1**. All absorbance values were compared to that of the PBS control.

Combinational effect analysis and dose-effect-based median-effect principle were also calculated as described below. Shortly, in the combinational effect analysis, having a combinatorial index (CI) CI>1 indicates a positive and a CI>1 a negative effect. For the dose-effect-based median-effect principle, a CI<0.7 indicates synergism, a 0.7<CI>1.45 indicates an additive effect, while a CI>1.45 indicates an antagonistic effect. The WST-1 assays were also complemented with assessment of cell confluence, cytotoxicity, and apoptosis using the IncuCyte S3 Live−Cell Analysis System, and the data extrapolated from these assays are presented below.

### Single Inhibitor Treatments

BYL719 induced dose-dependent effects in both DAOY and UW228-3. The highest BYL719 dose induced a significant decrease in absorbance compared to PBS at all timepoints for DAOY (for all at least p<0.05), while this was the case for only the 5 μM dose at 48 h for UW228-3 (p<0.05) (**Figures 1A, F**, respectively).

JNJ-42756493 also induced dose-dependent effects in DAOY and UW228-3, but was less potent in UW228-3. Its highest dose induced a significant decrease in absorbance as compared to PBS at all timepoints in both lines (for all at least p<0.05), (**Figures 1B, G**, respectively).

PD-332991 decreased absorbance in DAOY with its highest concentration as compared to PBS at all timepoints (for all at least p<0.05), but not in UW228-3 (**Figures 1D, I**, respectively).

IC50 (inhibitory concentration 50%). To better evaluate the sensitivity of the two cell lines to the inhibitors, IC50 values for all cell lines were calculated and are presented in **Table 1**.

**Table 1:** Estimation of inhibitory concentration 50% (IC50) based on WST−1 viability analysis following treatment with the PI3K inhibitor (BYL719), the FGFR inhibitor (JNJ-42756493), the CDK4/6 inhibitor (PD-0332991), and the cytostatic drugs cisplatin and vincristine for 24, 48, and 72 h.

Drugs	Cell lines	*IC_50_ (μM)*		
		24h	48h	72h
BYL	DAOY	7.36	10.15 ^b^	5.65
	UW228-3	>10^a^	>10^a^	>10^a^
JNJ	DAOY	>10^a^	9.21	4.74
	UW228-3	>10^a^	>10^a^	>10^a^
PD	DAOY	20.57 ^b^	15.57	14.42
	UW228-3	>20^a^	>20^a^	>20^a^
CIS	DAOY	5.42	1.84	0.67
	UW228-3	>10^a^	>10^a^	10.35
VIN	DAOY	0.60	0.17	0.04
	UW228-3	NA^c^	0.98	0.83

The inhibitory concentration 50% (IC_50_) for each cell line for each drug was determined from log concentrations-effect curves in GraphPad Prism using non-linear regression analysis. ^a.^The IC_50_ was lower/higher the tested concentration. ^b.^The IC_50_ is slightly above concentration range. ^c.^Not applicable (NA): The IC_50_ could not be determined. BYL, BYL719; JNJ, JNJ-42756493; PD, PD-0332991; CIS, cisplatin; VIN, vincristine.

To summarize, the data indicated that DAOY was more sensitive than UW228-3 to all of the single inhibitor treatments.

### Inhibitor Combination Treatments

The highest BYL719 and JNJ-42756493 dose combination decreased viability at all timepoints in both DAOY and UW228-3, as did the intermediate dose combination at all timepoints for UW228-3 and at 48 h for DAOY, while with the lowest dose combination, this was the case only at 72 h for DAOY (for all at least p<0.05) (**Figures 1C, H**, respectively).

The highest BYL719 and PD-332991 dose combination significantly decreased viability at all timepoints in both DAOY and UW228-3 (for all at least p<0.05) (**Figures 1E, J**, respectively). Notably, in DAOY viability was **<**50**%** after 72 h for all doses, as was the case for UW228-3 with the highest doses.

Combining JNJ-42756493 and PD-332991 decreased viability significantly with most doses in both cell lines at most timepoints compared to PBS (for all at least p<0.05) (**Supplementary Figure 1**).

### High Single-Agent and Median-Effect After Inhibitor Combination Treatments

**Figure 2:**
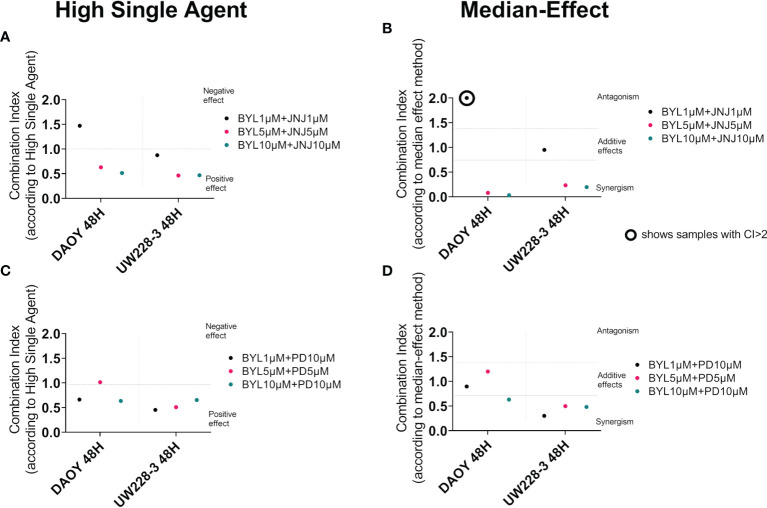
Combination treatments with BYL719 and JNJ-42756493 and BYL719 and PD-0332991 in MB cell lines DAOY and UW228-3. Combination index (CI) analysis was calculated in MB cell lines treated for 48 h by using the Highest Activity Agent method [**(A)** BYL719-JNJ-42756493 and **(C)** BYL719-PD-0332991] and the median-effect method **(B, D)**. In A and C charts, CI<1 indicates positive combination effect and CI>1 negative effect, while in B and D, CI<0.7 suggests synergy, CI>1.45 antagonism, and 0.7<CI>1.45 additive combinational effects. CIs were calculated from the means of three experiments, analyzed with WST-1.

To further evaluate positive or negative combinational effects as well as possible synergistic, additive, or antagonistic effects of the drug combinations, the effect-based Highest Single-Agent approach and dose-effect-based median-effect principle were used (31, 32). Combinational indexes (CIs) at 48 h after treatment of DAOY and UW228-3 with BYL719 and JNJ-42756493, and BYL719 and PD-0332991, are shown in **Figure 2**, and for JNJ-42756493 and PD-0332991 are presented in **Supplementary Figure 2**. The overall combination effects by the Highest Single-Agent Approach for the former two combinations were positive (CI<1) or neutral, with exception of the lowest BYL719 and JNJ-42756493 dose combination in DAOY (**Figures 2A, C**, respectively).

The median-effect principle indicated synergy in most BYL719 and JNJ-42756493 and BYL719 and PD-0332991 combinations except for the lowest BYL719 and JNJ-42756493 dose combination that was additive for UW228-3 and antagonistic for DAOY, and the lowest**/**middle dose BYL719 and PD-0332991 combinations which were additive in DAOY (**Figures 2B, D**, respectively). The JNJ-42756493 and PD-0332991 combinations had mainly positive effects on UW228-3, but negative effects on DAOY with the high single-agent approach (**Supplementary Figure 2A**). By the median-effect method, the median CI could not be calculated on DAOY due to the poor goodness-of-fit of both drugs (r<0.85), but on UW228-3, synergistic effects were observed (**Supplementary Figure 2B**).

To summarize, most drug combinations had notable very positive effects, especially in the resistant cell line UW228-3, where synergistic effects were recorded with both methods.

### Cell Confluence, Cytotoxicity, and Apoptosis of MB Cell Lines DAOY and UW228-3 Following Single and Combined Treatments With PI3K, FGFR, and CDK4/6 Inhibitors

**Figure 3:**
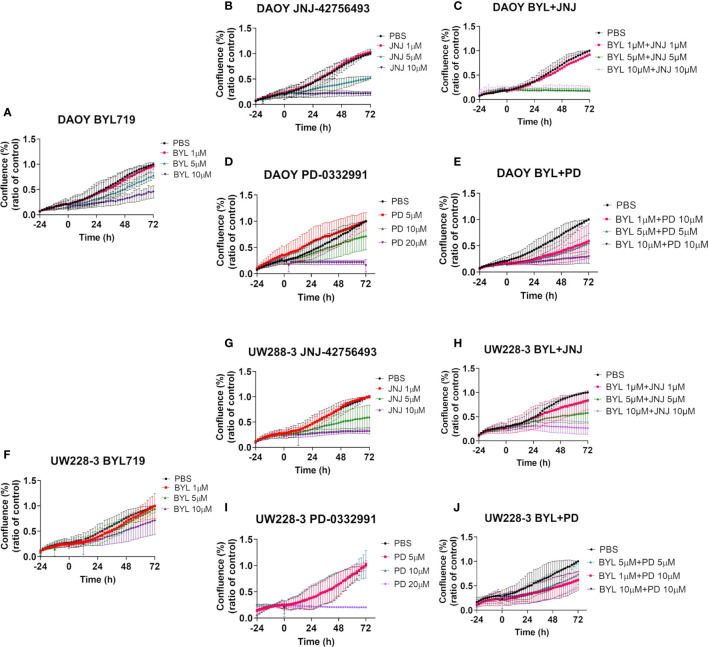
Cell confluence after single and combination treatments with BYL719 and JNJ-42756493 and BYL719 and PD-0332991 in MB cell lines DAOY and UW228-3. Single treatments are shown for BYL719 in **(A, F)** JNJ-42756493 in **(B, G)** and for PD-332991 in **(D, I)** for DAOY and UW228-3, respectively. Combination treatments of BYL719 and JNJ-42756493 are shown in **(C, H)** and of BYL719 and PD-332991 in **(E, J)** for DAOY and UW228-3, respectively. BYL, BYL719; JNJ, JNJ-42756493; PD, PD-0332991.

Cell confluence, cytotoxicity, and apoptosis of DAOY and UW228-3 were followed in the IncuCyte S3 Live-Cell Analysis system. Cell confluence data, compiled after three experiments, of DAOY and UW228-3, respectively, after single doses of BYL719 (1–10 µM), JNJ-42756493 (1–10 μM), or PD-0332991 (5–20 μM), respectively, and for combinations of BYL719, and JNJ-42756493 (both 1–10 µM) and PD-0331991 (5–10 µM) in comparison to PBS are depicted in **Figure 3**. Cytotoxicity (depicted in **Supplementary Figure 4**) and apoptosis were assayed in parallel (see below).

#### Effects With Single Inhibitor Treatments on Cell Confluence

The highest BYL719 dose inhibited an increase in cell confluence (when compared to PBS) in DAOY after 72 h, while the intermediate dose presented a marginal effect, and the lowest dose had no effect (**Figure 3A**). For UW228-3, only the highest dose resulted in a marginal effect after 72 h (**Figure 3F**).

The highest JNJ-42756493 inhibited an increase in the cell confluence (when compared to PBS) of both DAOY and UW228-3, while for both cell lines the intermediate dose had only a slight effect, while the lowest dose had no effect (**Figures 3B, G**, respectively).

The highest PD-0332991 significantly inhibited an increase in cell confluence the whole follow-up period (when compared to PBS) for both DAOY and UW228-3, and the intermediate dose had a marginal effect in DAOY, while no other effects were observed (**Figures 3D, I**, respectively).

#### Effects of Inhibitor Combination Treatments on Cell Confluence

Combinations of the two highest doses of BYL719 and JNJ-42756493 inhibited an increase in cell confluence completely (when compared to PBS) of DAOY, as was the case for UW228-3 with the highest dose combination (**Figures 3C, H**, respectively). For DAOY no effect was observed with the lowest dose combination, while for UW228-3 intermediate effects were observed with the two lower dose combinations (**Figures 3C, H**, respectively).

Combining 10 and 10 µM, and 5 and 5 µM of BYL719 with PD-332991, respectively, inhibited an increase in cell confluence at all timepoints (when compared to PBS) for DAOY, while for UW228-3, a similar but less prominent tendency was observed when compared to the use of the corresponding single drug doses (**Figures 3E, J**, respectively).

Combining JNJ-42756493 and PD-332991 (when compared to PBS) slightly improved the inhibition of an increase in cell confluence as compared to using each single drug alone in both cell lines (**Supplementary Figure 3**).

#### Cytotoxic and Apoptotic Effects of Single Inhibitor Treatments

After BYL719 treatment, no obvious cytotoxicity was observed in DAOY or UW228-3 except for a minimal effect with the highest dose in DAOY (**Supplementary Figures 4A, G**, respectively).

Only the highest JNJ-42756493 dose induced a marginal cytotoxic effect in DAOY and UW228-3, while for all others no, major effects were observed (**Supplementary Figures 4B, H**, respectively).

The highest PD-0332991 dose induced considerable cytotoxicity in both DAOY and UW228-3 (**Supplementary Figures 4C, I**, respectively).

Analogous effects were observed upon evaluating apoptosis (data not shown).

#### Cytotoxic and Apoptotic Effects of Inhibitor Combinational Treatments

Combining the highest BYL719 and JNJ-42756493 doses resulted in pronounced cytotoxicity in DAOY and some cytotoxicity in UW228-3, while intermediate doses gave some cytotoxicity in DAOY, while all other effects were marginal (**Supplementary Figures 4D, J**, respectively).

Combining the two highest BYL719 and PD-332991 combination doses induced some marginal cytotoxicity in both DAOY and UW228-3 as compared to the use of one single drug at the same concentration (**Supplementary Figures 4E, K**, respectively).

Combining JNJ-42756493 and PD-332991 induced some marginal cytotoxicity in DAOY, but not in UW228-3 at all doses (**Supplementary Figures 4F, L**, respectively).

Analogous effects were obtained when evaluating apoptosis (data not shown).

To summarize, inhibition of the increase in cell confluence paralleled the inhibition of viability for both cell lines. More limited effects were obtained with regard to cytotoxicity and apoptosis, with the exception of when high doses of PD-0332991 were used and to a lesser extent when BYL719 and JNJ-42756493 were combined.

### Viability After Single and Combined Treatments With Cisplatin and Vincristine and Upon Combination With PI3K, FGFR, and CDK4/6 Inhibitors BYL719, JNJ-42756493, and PD-0332991, Respectively, in MB Cell Lines DAOY and UW228-3

Data from at least three experiments with WST-1 viability assays 24, 48, and 72 h after single administrations of cisplatin (1–10 µM) and vincristine (5–100 nM) are depicted below, and with regard to IC50 values in **Table 1** above. Moreover, at least three experiments with WST-1 assays with combinations of either cisplatin 1–10 µM or vincristine 5 nM–1 µM with either BYL719, 1.0–10 µM, or JNJ-42756493, 1.0–10.0 μM or PD-0332991, 5.0–20 μM, for 24, 48, and 72 h are also described below. In addition, calculations according to the combinational effect analysis and dose-effect-based median-effect principle were done. The effects on viability using WST-1 assays were also complemented with the analysis of proliferation, cytotoxicity, and apoptosis using the IncuCyte S3 Live−Cell Analysis System, and the data extrapolated from these assays are presented below.

### Single CT Treatments

**Figure 4:**
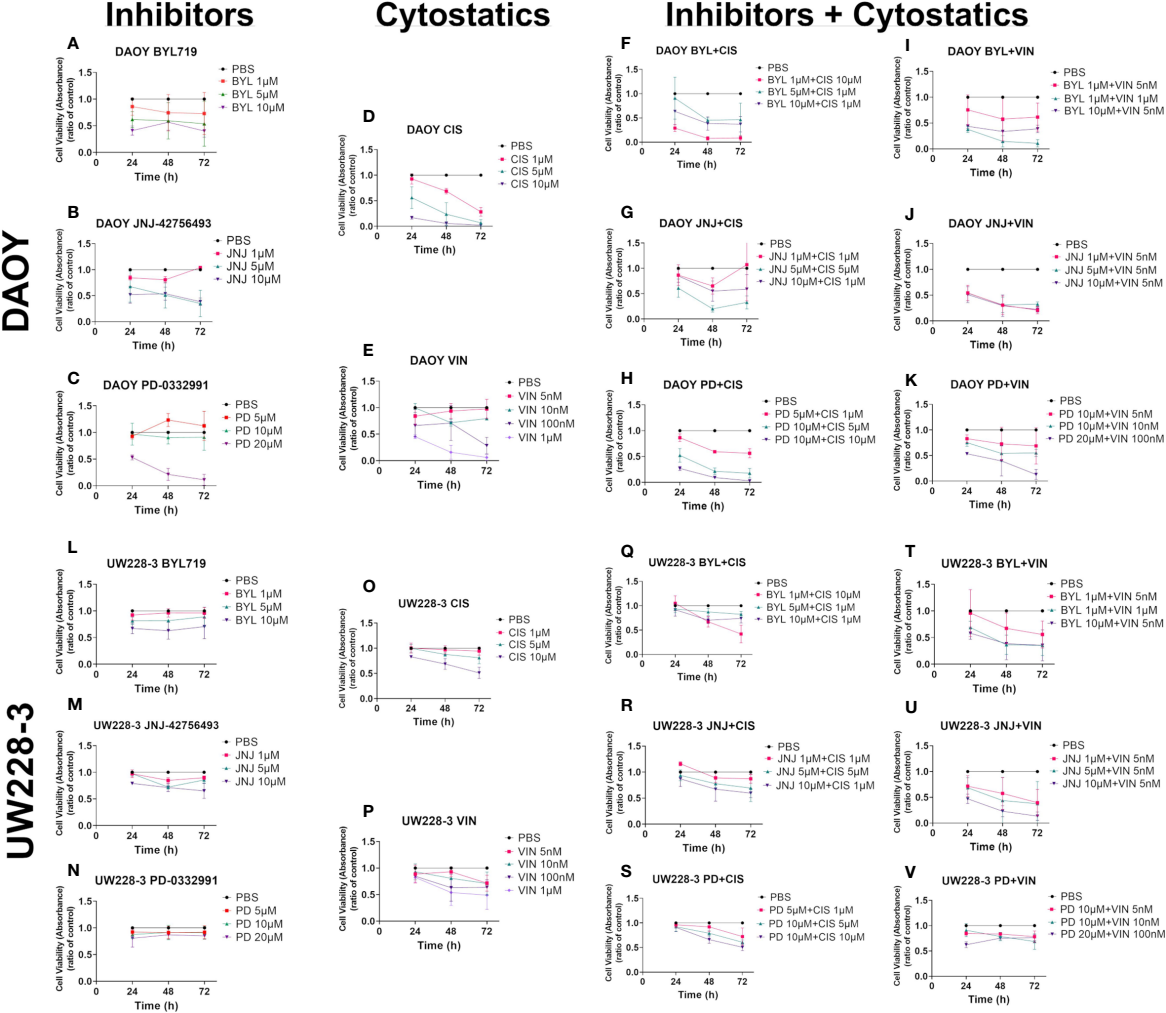
WST-1 viability assays after single and combinational treatment of BYL719, JNJ-42756493, and PD-0332991, respectively, with cisplatin or vincristine respectively on DAOY or UW228-3. Viability analysis measured as absorbance, after treatment of DAOY and UW228-3 respectively, for 24, 48, and 72 h with BYL719 **(A, L)**, JNJ-42756493 **(B, M)**; and PD-0332991 **(C, N)**, Cisplatin **(D, O)**, and Vincristine **(E, P)**, respectively. Combinational treatments are shown for DAOY **(F–K)** and UW228-3 **(Q–V)**. The graphs represent mean values ± standard deviation (SD) from three experimental runs per cell line. BYL, BYL719; JNJ, JNJ-42756493; PD, PD-0332991; CIS, cisplatin; VIN, vincristine.

Cisplatin induced dose-dependent responses, with DAOY being more sensitive than UW228-3 with both presenting significantly decreased viability compared to PBS with the highest dose the whole observation period, while only DAOY presented a significant decrease in viability at 72 h after treatment with the lower doses (for all at least p<0.05) (**Figures 4D, O**, respectively).Vincristine induced dose-dependent responses, with DAOY being more sensitive than UW228-3 and showing a statistically significant decrease in viability as compared to PBS at all timepoints with the highest dose, and after 72 h with both lower doses (for all at least p<0.05) (**Figure 4E**). For UW228-3, significance was observed only in sporadic cases (**Figure 4P**).

IC50 values were calculated to better evaluate the sensitivity of the two cell lines, and in **Table 1**, the data clearly indicate DAOY was more sensitive than UW228-3 to both cisplatin and vincristine.

### Inhibitor and CT Combination Treatments

Data combining BYL719 or JNJ-42756493 or PD-0332991 with either cisplatin or vincristine for treatment of DAOY and UW228-3, as well as single treatments with all drugs for comparison, are shown in **Figure 4**. The same dose combinations were used for both DAOY and UW228-3; however, for the sake of simplicity, only three dose combinations of each category are shown in **Figure 4**, despite the fact that more combinations were examined. This has resulted in that there are some differences in the doses used for BYL719 and PD-0332991 when combined with cisplatin as compared to vincristine, while for JNJ-42756493, the same concentrations were used in combination with both cisplatin and vincristine (**Figure 4**).

Combining BYL719 and cisplatin gave dose-dependent responses in both DAOY and UW228-3, with a significant decrease in viability compared to PBS for most combinations after 48 and 72 h in DAOY and for the 10 µM BYL719 and 1 µM cisplatin combination after 48 h in UW228-3 (for all at least p<0.05) (**Figures 4F, Q**, respectively).

Combining BYL719 and vincristine gave dose-dependent responses in both DAOY and UW228-3, but significant decreases in viability compared to PBS was observed for all timepoints only in DAOY except for with the lowest dose BYL719 and vincristine combination (for all at least p<0.05) (**Figure 4I, T**, respectively).

Combining JNJ-42756493 and cisplatin gave dose-dependent responses. A significant decrease in viability compared to PBS was observed for 5 µM JNJ-42756493 and 5 µM cisplatin at 48 and 72 h for DAOY, and with 10 µM JNJ-42756493 and 1 µM cisplatin after 72 h for both DAOY and UW228-3 (for all at least p<0.05) (**Figures 4G, R**, respectively).

Combining JNJ-42756493 and vincristine gave dose-dependent responses and a significant decrease in viability compared to PBS for most dose combinations at all timepoints for DAOY (except for 10 µM JNJ-42756493 and 5 nM vincristine at 24 and 48 h) and for UW228-3 with the highest JNJ-42756493 and vincristine combination (for all at least p<0.05) (**Figures 4J, U**, respectively).

Combining PD-0332991 and cisplatin gave dose-dependent responses and a significant decrease in viability compared to PBS for almost all dose combinations at all timepoints for DAOY, and after 48 and 72 h for all combinations (except for 5 µM PD-0332991 and 1 µM cisplatin) for UW228-3 (for all at least p<0.05) (**Figures 4H, S**, respectively).

Combining PD-0332991 and vincristine gave dose-dependent responses and a significant decrease in viability compared to PBS for all combinations at all timepoints except at 48 h with 20 µM PD-0332991 and 100 nM vincristine and the lowest combination for DAOY, while for UW228-3, only sporadic significant effects were observed with the different combinations and timepoints (for all at least p<0.05) (**Figures 4K, V**, respectively).

### Calculations of CIs After Inhibitor Combination Treatments

Combination indexes (CIs) of the inhibitors BYL719, JNJ-42756493, or PD-0332991 combined with cisplatin or vincristine were calculated 24 and 48 h after treatment of DAOY and UW228-3, and the data after 48 h treatment are shown in **Supplementary Figure 5**.

The overall combination effects show some variations with synergistic, additive, and antagonistic effects depending both on the specific drug and cell line. Treatment including cisplatin combinations showed a more complex picture than those including vincristine combinations.

More specifically, combining cisplatin with BYL719 or JNJ-42756493 mainly gave additive or synergistic effects for DAOY, while for UW228-3, mainly neutral or antagonistic effects were observed, while upon combining cisplatin and PD-0332991, more neutral effects were obtained for both cell lines (**Supplementary Figures 5A–F**). When combining vincristine with BYL719 or JNJ-42756493 or PD-0332991, mainly additive or synergistic effects (when possible to calculate) were observed for DAOY, and this was also mainly the case (and especially in combination with BYL719) for UW228-3 (**Supplementary Figures 5G–L**, respectively).

To summarize, most PI3K, FGFR, and CDK4/6 combinations with vincristine showed synergistic or additive or synergistic effects on both DAOY and UW228-3, while their combinations with cisplatin were more complex and showed mainly additive effects for DAOY but not for UW228-3.

### Cell Confluence, Cytotoxicity, and Apoptosis of DAOY and UW228-3 After Single and Combined Treatments With PI3K, FGFR, CDK4/6 Inhibitors BYL719, JNJ-42756493, and PD-0332991, Respectively, and Cisplatin or Vincristine

**Figure 5:**
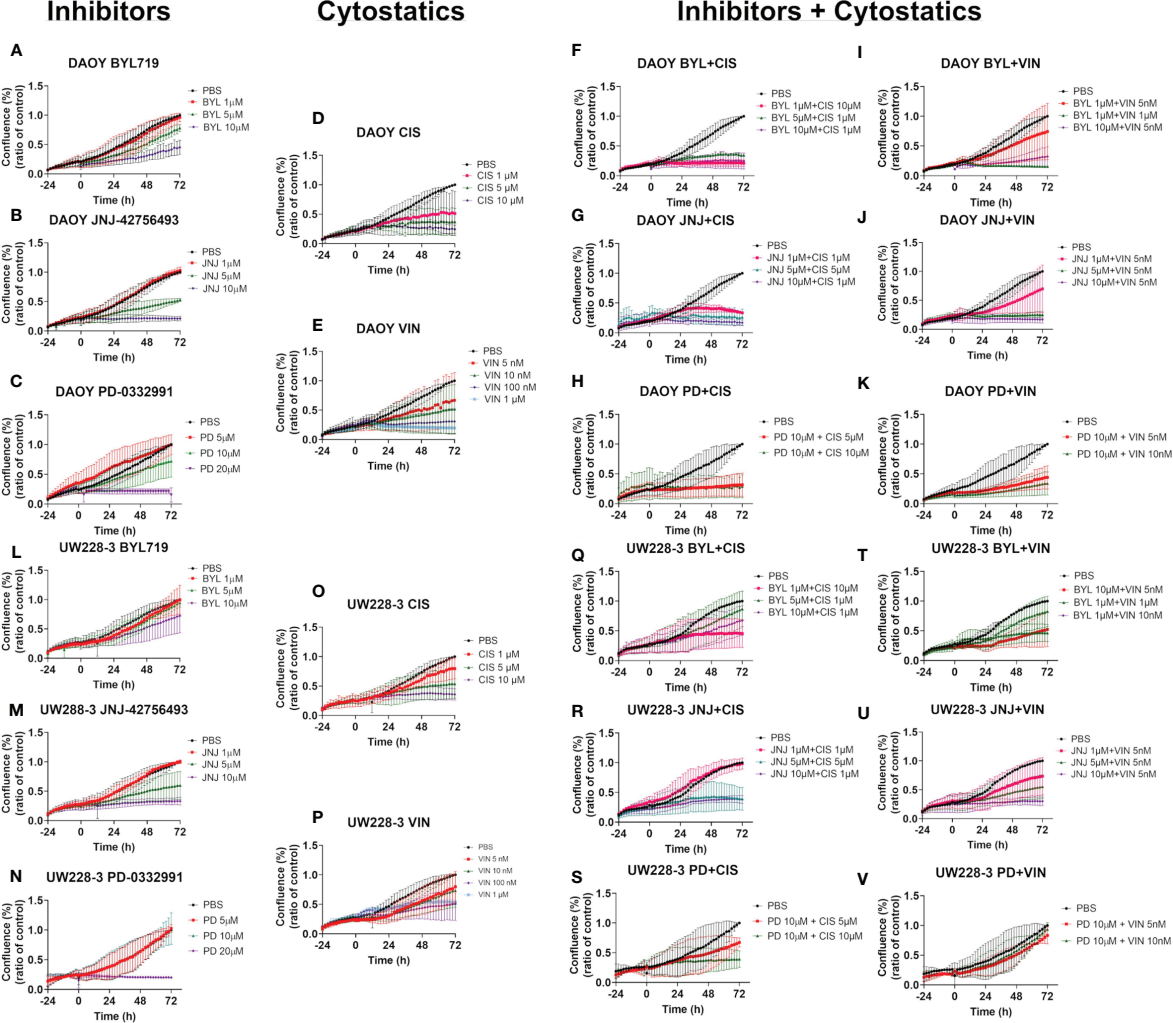
Cell confluence after single and combination treatments with BYL719, JNJ-42756493, and PD-0332991 and cisplatin or vincristine on MB cell lines DAOY and UW228-3. Single treatments of DAOY and UW228-3, respectively, are shown for BYL719 in **(A, L)**; JNJ-42756493 in **(B, M)**; and for PD-332991 in **(C, N)**; cisplatin in **(D, O)** and vincristine in **(E, P)**, respectively. Corresponding combination treatments of DAOY and UW228-3, respectively, with BYL719 and cytostatics are shown in **(F, I, Q, T)**; with JNJ-42756493 and cytostatics in **(G, J, R, U)**; and with PD-332991 and cytostatics in **(H, K, S, V)**, respectively. BYL, BYL719; JNJ, JNJ-42756493; PD, PD-0332991; CIS, cisplatin; VIN, vincristine.

Changes in cell confluence, cytotoxicity, and apoptosis of DAOY and UW228-3 were followed in the IncuCyte S3 Live-Cell Analysis system. Effects on cell confluence compiled after at least three experiments on DAOY and UW228-3 after treatment with single doses of cisplatin (1–10 µM) and vincristine (5 nM–1 μM), BYL719 and JNJ-42756493 (both 1–10 µM), or PD-0332991 (5–20 μM) and their combinations in comparison to PBS are shown in **Figure 5**.

In parallel, at least three experiments were also performed for cytotoxicity, but here only a representative figure for cytotoxicity is presented below. Apoptosis experiments were performed once, since no major effects were observed (data not shown).

#### Effects of Single CT Treatments on Cell Confluence

The two highest doses of cisplatin induced an almost complete inhibition of an increase in cell confluence (when compared to PBS) in DAOY, and this was also the case with the highest cisplatin dose for UW228-3, while the lower doses only gave slight inhibition (**Figures 5D, O**, respectively).

Dose-dependent inhibition of increase in cell confluence (when compared to PBS) was observed upon treatment of DAOY and UW228-3 with vincristine, and complete inhibition of proliferation was observed with the two highest doses for DAOY, while only intermediate effects were obtained for UW228-3 with the corresponding highest doses (**Figures 5E, P**, respectively).

For BYL719, JNJ-42756493, and PD-332991, please see the corresponding text above (**Figure 3**).

#### Effects of Inhibitor and CT Combination Treatments on Cell Confluence

Combining BYL719 and cisplatin gave dose-dependent responses in both DAOY and UW228-3, with a complete inhibition of increased cell confluence (when compared to PBS) for all combinations for DAOY, while for UW228-3, this was only the case for the combination including the highest cisplatin concentration (**Figures 5F, Q**, respectively).

Combining BYL719 and vincristine gave dose-dependent responses in DAOY and UW228-3, with a complete inhibition of increased cell confluence (when compared to PBS) for the highest dose combinations in DAOY and UW228-3 and intermediate decreases with the other doses (**Figures 5I, T**, respectively).

Combining JNJ-42756493 and cisplatin gave dose-dependent responses and a complete inhibition of increased cell confluence (when compared to PBS) for all dose combinations for both DAOY and UW228-3 (**Figures 5G, R**, respectively).

Combining JNJ-42756493 and vincristine gave dose-dependent responses and complete inhibition of increased cell confluence (when compared to PBS) within all dose combinations at all timepoints for both DAOY and UW228-3 (**Figures 5J, U**, respectively).

Combining PD-0332991 and cisplatin gave dose-dependent responses and a complete inhibition of increased cell confluence (when compared to PBS) for both dose combinations at all timepoints for DAOY, and with the highest dose combination for UW228-3 (**Figures 5H, S**, respectively).

Combining PD-0332991 and vincristine gave dose-dependent responses and a complete inhibition of increased cell confluence (when compared to PBS) for both dose combinations at all timepoints for DAOY, but only to some extent for UW228-3 (**Figures 5J, V**, respectively).

#### Effects of Single CT Treatments on Cytotoxicity and Apoptosis

Cisplatin and vincristine. Major cytotoxic effects were not observed with cisplatin or vincristine alone with the doses used on DAOY or UW228-3 (**Supplementary Figures 6D, E, O, P**, respectively). Analogous data were shown for apoptosis with a marginal increase in apoptosis in DAOY when using the highest cisplatin and vincristine doses (data not shown).

For BYL719, JNJ-42756493, and PD-332991 please see the corresponding text above (**Supplementary Figure 4**).

#### Combination of Inhibitor and CT Treatments and Cytotoxicity and Apoptosis

Combining BYL719 and cisplatin induced only some marked cytotoxicity when using the 1 μM BYL719 and 10 μM cisplatin combination as compared to the effects of each drug alone in DAOY, while there was no effect whatsoever in UW228-3 (**Supplementary Figures 6F, Q**, respectively).

Combining BYL719 and vincristine resulted in increased cytotoxicity, especially with the highest dose combination as compared to the corresponding single treatments in DAOY, while no major effects were observed in UW228-3 (**Supplementary Figures 6I, T**, respectively).

Combining JNJ-42756493 and cisplatin induced some marginal cytotoxic effects with the two highest drug doses as compared to the corresponding single treatments in DAOY, but not in UW228-3 (**Supplementary Figures 6G, R**, respectively).

Combining JNJ-42756493 and vincristine induced some marginal cytotoxic effects with the highest dose combinations as compared to the corresponding single treatments in DAOY, but not in UW228-3 (**Supplementary Figures 6J, U**, respectively).

Combining PD-0332991 and cisplatin did not improve any cytotoxic effects as compared to the single treatments for either DAOY or UW228-3 (**Supplementary Figures 6H, S**, respectively).

Combining PD-0332991 and vincristine did not improve any cytotoxic effects as compared to the single treatments for either DAOY or UW228-3 (**Supplementary Figures 6K, V**, respectively).

Analogous trends with the weak combinational cytotoxic effects were observed with regard to apoptosis (data not shown).

In summary, DAOY was more sensitive than UW228-3 when treated with inhibitors and cytostatic drugs. Inhibition of the increase in cell confluence when compared to PBS paralleled inhibition of viability for both cell lines, while limited effects, with few exceptions on DAOY, were obtained with regard to cytotoxicity and apoptosis upon combining the cytostatic drugs with the inhibitors.

### Viability After Single and Combined Treatments With Irradiation of 0, 2, and 10 Gray and Upon Combination With PI3K, FGFR, and CDK4/6 Inhibitors in MB Cell Lines DAOY and UW228-3

**Figure 6:**
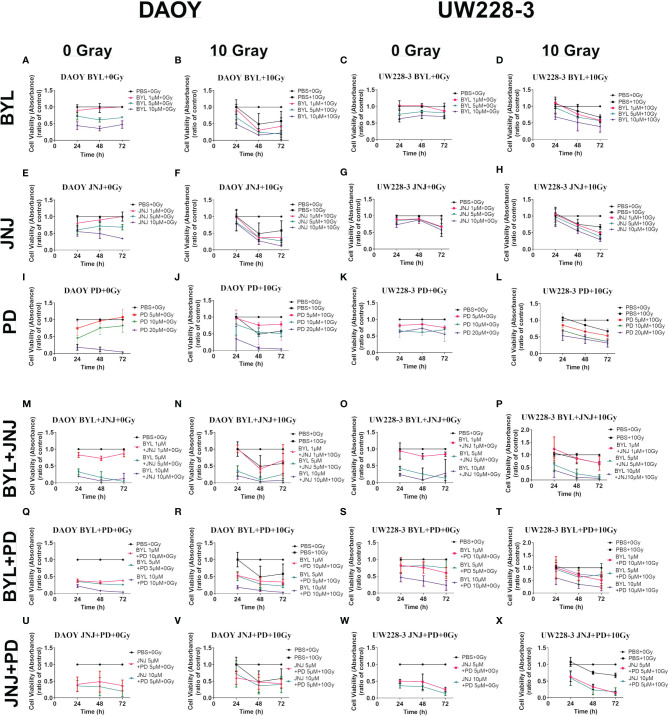
WST-1 viability assays of the MB cell lines DAOY and UW228-3 first treated with single or combinational treatment of PI3K, FGFR, and CDK4/6 inhibitors BYL719, JNJ-42756493, and PD-0332991, respectively, and then not irradiated (0 Gy) or irradiated 3 h later with 10 Gy. Viability analysis measured as absorbance, after treatment for 24, 48, and 72 h with BYL719 **(A–D)**, JNJ-42756493 **(E–H)**; and PD-0332991 **(I–L)**, of DAOY and UW228-3, respectively. Their combinations are shown for DAOY **(M, N, Q, R, U, V)** and for UW228-3 **(O, P, S, T, W, X)**. BYL, BYL719; JNJ, JNJ-42756493; PD, PD-0332991; Gy, Gray; MB, medulloblastoma.

Data from at least three experiments with WST-1 viability assays 24, 48, and 72 h after treatment of DAOY and UW228-3, respectively, with BYL719, JNJ-42756493, and PD-0332991 followed by no irradiation, or 10 Gray (Gy); or no irradiation, and 2 Gy are depicted in **Figure 6** and **Supplementary Figure 7**, respectively. In addition, combinations of BYL719, JNJ-42756493 (both 1–10 μM), or PD-0332991 (0.1–5 μM) with no irradiation or with 10, or no irradiation and 2 Gy, respectively, for 24, 48, and 72 h after the treatments were also performed and shown in **Figure 6** and **Supplementary Figure 7**, respectively.

#### Single Inhibitor Treatments With Irradiation

Single treatments with 2 and 10 Gy did not affect viability of DAOY but did significantly affect viability of UW228-3 at 72 h (for all at least p<0.05) (**Supplementary Figures 7A, B** and **Figures 6A–D**, respectively).

Combining 5 and 10 μM of BYL719 with 2 or 10 Gy significantly reduced viability in both DAOY and UW228-3 at almost all timepoints (for all at least p<0.05) (**Supplementary Figures 7A, B** and **Figures 6B, D**, respectively). Combining 1 μM BYL719 with 2 or 10 Gy also significantly reduced cell viability of UW228-3 after 48 and 72 h, while for DAOY, only the combination with 10 Gy was effective with the latter BYL719 dose after 48 and 72 h (for all at least p<0.05) (**Supplementary Figures 7A, B** and **Figures 6B, D**, respectively).

Combining 5 and 10 μM of JNJ-42756493, with 2 or 10 Gy significantly reduced viability in both DAOY and UW228-3 after 48 and 72 h (for all at least p<0.05) (**Supplementary Figures 7C, D** and **Figures 6F, H**, respectively). In addition, combining 1 μM JNJ-42756493 with 2 or 10 Gy significantly reduced cell viability of UW228-3 after 48 and 72 h of drug treatment, while for DAOY only the combination with 10 Gy dose with the latter JNJ-42756493 dose was effective after 48 and 72 h (for all at least p<0.05) (**Supplementary Figures 7C, D** and **Figures 6F, H**, respectively).

The combination of all concentrations of CDK4/6 inhibitor, PD-332991, with both 2 or 10 Gy significantly decreased cell viability at almost all timepoints for both DAOY and UW228-3 (for all at least p<0.05) (**Supplementary Figures 7E, F** and **Figures 6J, L**, respectively).

To summarize, adding 10 Gy to single treatments of BYL719, JNJ-42756493, or PD-332991 tended to improve inhibition of viability of DAOY and in particular of UW228-3, especially at the 72 h timepoint.

#### Combination of Inhibitor Treatments and Irradiation

Combining BYL719 and JNJ-42756493 with 2 or 10 Gy significantly decreased cell viability at all doses and all timepoints in DAOY, as was also the case for UW228-3 for most timepoints and combinations except for the lowest combination (1 μM BYL719 and 1 μM JNJ-42756493 with both 2 and 10 Gy, respectively) (for all at least p<0.05) (**Supplementary Figures 7G, H** and **Figures 6N, P**, respectively). However, the effects of the combinational inhibitor treatments were not enhanced in UW228-3 by adding irradiation (**Supplementary Figure 7H** and **Figure 6P**, respectively).

Combining BYL719 and PD-332991 with 2 and 10 Gy significantly decreased cell viability at all concentrations and all timepoints in DAOY, while this was the case for UW228-3 only with the highest inhibitor concentrations with both 2 and 10 Gy (for all at least p<0.05) (**Supplementary Figures 7I, J** and **Figures 6R, T**, respectively). However, the effects of the combinational inhibitor treatments were not enhanced by irradiation in UW228-3 (**Supplementary Figure 7J** and **Figure 6T**, respectively).

Combining JNJ-42756493 with PD-332991 with 2 and 10 Gy significantly reduced viability at all timepoints with all combinations in both DAOY and UW228-3 (for all at least p<0.05) (**Supplementary Figures 7K, L** and **Figures 6V, X**, respectively). However, during the observation period, the effects of the combinational inhibitor treatments were not enhanced by adding irradiation treatment (**Supplementary Figures 7K, L** and **Figures 6V, X**, respectively).

To summarize, adding RT to the already beneficial combinations of the inhibitors maintained but did not increase the enhanced inhibition of the inhibitors on either DAOY or UW228-3.

## Discussion

In this study, the PI3K inhibitor alpelisib (BYL719), the FGFR inhibitor erdafitinib (JNJ-42756493), and the CDK4/6 inhibitor palbociclib (PD-332991) were tested alone and combined or together with cisplatin, vincristine, or RT for their ability to inhibit the growth of SHH MB cell lines DAOY and UW228-3.

Both DAOY and UW228-3, but the latter to a lower extent, showed dose-dependent decreases in viability and proliferation upon treatment with BYL719, JNJ-42756493, and PD-332991, cisplatin and vincristine, but only PD-332991 induced pronounced effects on cytotoxicity or apoptosis in the highest tested dose. Upon combining the inhibitors, additive/synergistic effects were observed on the decrease of viability and especially of the more resistant cell line UW228-3, as did the inhibition of an increase in cell confluence when compared to the PBS control. Likewise, upon combining the inhibitors with cisplatin or vincristine, some positive effects were observed, and the most prominent (additive/synergistic) effects on viability and cell confluence were observed when combining BYL719 and cisplatin or vincristine. Finally, both DAOY and UW228-3 were exposed to the inhibitors alone or combined, with and without irradiation with 2 or 10 Gy. Some positive effects were obtained with irradiation with the administration of inhibitors alone, but irradiation did not increase the efficacy when two inhibitors were combined.

Others and we have previously reported that MB cell lines respond with decreased viability to PI3K inhibitors such as BEZ235, BKM120, and GDC-0941 and CDK4/6 inhibitors such SCH727965, and PD-332991, but only we have shown MB cell line sensitivity to FGFR inhibitors such as AZD4547 (26, 29, 33–35). In addition, the influence of CDK4/6 and CD4/6 inhibitors on MB has been studied more specifically, and beneficial effects of the inhibitors have been reported (36, 37). However, to our knowledge, there are no previous studies combining PI3K, FGFR, and CDK4/6 inhibitors, and especially not the more recently FDA-approved inhibitors (BYL719, JNJ-42756493, and PD-332991, respectively) in MB cell lines, so our combination treatment data could be potentially promising for future MB treatment. Moreover, the possibility to use lower concentrations of the inhibitors when combining them, and obtaining synergy and improved antitumor efficacy, may also result in reduced side effects. In addition, targeting MB with two different inhibitors might reduce the risk of drug resistance.

Remarkably, although marked inhibition of viability and an inhibition in increase in cell confluence when compared to the PBS control were obtained in both cell lines upon treatment with most of the single and combined inhibitor treatments, the effects on cytotoxicity and apoptosis were not as prominent. However, there were some exceptions, and when using PD-332991 or combining BYL719 and JNJ-42756493 in relatively high doses, cytotoxicity and apoptosis were observed. As mentioned above, there are limited or no reports on the use of the above drugs on MB cell lines; however, it has been reported previously that other PI3K inhibitors (BEZ235 and BYL719) do not have a major cytotoxic effect, while the cytotoxic effect of FGFR inhibitor AZD4547 is possibly somewhat increased (29).

The fact that the CDK4/6 inhibitor and the combination of BYL719 and JNJ-42756493 in high doses can induce cytotoxicity and apoptosis could be useful in clinical practice in MB patients; however, it is important to fine-tune the drug doses and use as low drug concentrations as possible in order to avoid side effects of the various drugs.

Notably, the combinational treatments of the PI3K inhibitors with either an FGFR or a CDK4/6 inhibitor were more prominent than the FGFR and CDK4/6 inhibitor combination in both MB cell lines. This could have been anticipated and depend on that both FGFR and CDK4/6 are associated with the PI3K pathway, so by combining them with a PI3K inhibitor may naturally enhance the effects on the PI3K pathway. Moreover, CDK4/6-related signaling molecules are downstream of most signaling pathways, such as the RAS pathway, the PI3K pathway, the TGF-β pathway, the p53 pathway, the Notch pathway, as well as the Myc pathway, suggesting that CDK4/6 blockade may inhibit these related signaling pathways to some extent as well (38). Unlike CDK4/6, FGFR on the other hand is linked upstream to the PI3K pathway (39, 40).

DAOY was generally more sensitive than UW228-3 to most treatments, which is in line with a previous report (29). However, the marked sensitivity of DAOY to the PI3K inhibitor in the viability tests could be explained as due DAOY having an in-frame deletion with a non-conserving PIK3R1 mutation (gene bank https://depmap.org/portal/). Nevertheless, the data described above underline that both MB cell lines, both exhibiting TP53 mutations, independently of having or not PI3K, CDK4/6, or FGFR mutations, can be sensitive to the tested inhibitors. This is in line with previous studies by others and us, where different tumors and tumor cell lines have been reported to be sensitive to PI3K and FGFR inhibitors, despite not having PI3K, CDK4/6, and or FGFR mutations (28, 29, 41). Exactly why UW228-3 is less but still quite sensitive to the combination treatments as compared to DAOY has not yet been explored in detail. However, the data obtained here were analogous with data in other reports by us on other cell lines, where the cell lines present differential responses often associated with their general sensitivity to CT and RT (41, 42). Additional studies would be required to elucidate the influence of the different inhibitors on specific mechanisms of action on the signaling pathways of the different MB cell lines.

As mentioned above, CT is included in current MB treatment, and the responses to CT may vary, as does the development of chemoresistance. Recently, similar and slightly different attempts as compared to ours have included the use of inhibitors in MB cell lines (34, 43, 44). In one of those studies, the hedgehog (HH) inhibitor, Vismodegib, was combined with the PI3K inhibitor, BEZ235, and cisplatin, and enhanced effects were also obtained (43). Thus, there are a plethora of possibilities that need to be investigated further. Hopefully, some of those combinations will overcome resistance development and possibly be able to reduce the doses of the included drugs, which in this study were similar to corresponding doses of the inhibitors and drugs assessed previously by others and us (29, 30, 34, 43–46).

Using irradiation resulted in some additional effects on the decrease of viability induced by single treatments with the inhibitors, but not when the inhibitors were combined. To our knowledge, there are limited studies combining the above inhibitors with irradiation. However, similar to us, one study reported that CDK4/6 inhibitors can enhance the radiosensitivity in multiple cell lines including MB cell lines (45, 46).

There are some limitations in the current study, since only two MB cell lines were examined and they both were derived from one specific group (SHH group). In the future, it would be valuable to pursue similar studies in a broader range of MB cell lines and under more strict conditions, e.g., to maintain SHH-signaling (47). In addition, only a limited number of drug concentrations were used, and also additional irradiation-drug treatment conditions could have been explored. In the future, also the range of drugs and irradiation doses and in which time ranges they should be distributed should be pursued further. Nevertheless, although PI3K and CDK4/6 inhibitors have been shown to have some effect on MB cell lines, to our knowledge, this is the first time that inhibitors against PI3K, CDK4/6, and FGFR were assessed in combination and with/without CT and with/without RT for treatment of MB cell lines.

To conclude, combination treatments of PI3K, FGFR, and/or CDK4/6 inhibitors, as well as especially the PI3K inhibitor BYL719 with cisplatin or vincristine on the MB cell lines DAOY and UW228-3, enhanced inhibition of cell viability and increase in cell confluence. In addition, irradiation of the corresponding cell lines tended to increase the effects of single but not combined inhibitor treatments. DAOY was generally more sensitive to the inhibitors compared to UW228-3, which could possibly be due to it having a PIK3R1 mutation, but further studies are needed to elucidate if indeed this is the case.

Taken together our data suggest that PI3K and FGFR or CDK4/6 inhibitor combinations or single inhibitor CT/RT combinations may provide possible therapeutic opportunities for therapy of resistant MBs and should be investigated further.

## Data Availability Statement

The original contributions presented in the study are included in the article/**Supplementary Material**. Further inquiries can be directed to the corresponding author.

## Author Contributions

ML and OK did the majority of the experiments, interpreted the data, calculated the statistics, and contributed to the writing of the manuscript. HM and EP-S collaborated with OK and performed some experiments, contributed together with OK in the graphs of the manuscript. SH and ER initiated the experiments and the interpretation of the initial experiments and contributed to the writing of the Material and Methods section, all under the supervision of OK. ML, TD, and OK made substantial contributions to conception and design, acquisition of data, analysis and interpretation of data and have been involved in drafting the manuscript and revising it critically for important intellectual content. TD has also provided the sources of the performance of the experiments. TD and OK provided the financial support for conducting the research project. All authors contributed to the article and approved the submitted version.

## Funding

This research was funded by Swedish Childhood Cancer Fond (PR2017-0042, PR2017-0052), the Swedish Cancer Society (180440, 2017/658), the Stockholm Cancer Society (181053), the Swedish Cancer and Allergy Foundation (190), Karolinska Institutet of Sweden (2018:0007), Stiftelsen AnnaBrita and Bo Casters Minne (LA2019-0080), Svenska Läkaresällskapet (SLS-934161), Åke wiberg foundation (M20-0061).

## Conflict of Interest

The authors declare that the research was conducted in the absence of any commercial or financial relationships that could be construed as a potential conflict of interest.

## Publisher’s Note

All claims expressed in this article are solely those of the authors and do not necessarily represent those of their affiliated organizations, or those of the publisher, the editors and the reviewers. Any product that may be evaluated in this article, or claim that may be made by its manufacturer, is not guaranteed or endorsed by the publisher.
