# Supplementary material for: Targeting PI3K, FGFR, CDK4/6 Signaling Pathways Together With Cytostatics and Radiotherapy in Two Medulloblastoma Cell Lines
Source: Front Oncol. 2021 Sep 24;11:748657. doi: 10.3389/fonc.2021.748657 (PMC8497987; doi:10.3389/fonc.2021.748657)
Supplement: Supplementary file 1 [file DataSheet_1.docx]

Supplementary Material

# Supplementary Figures


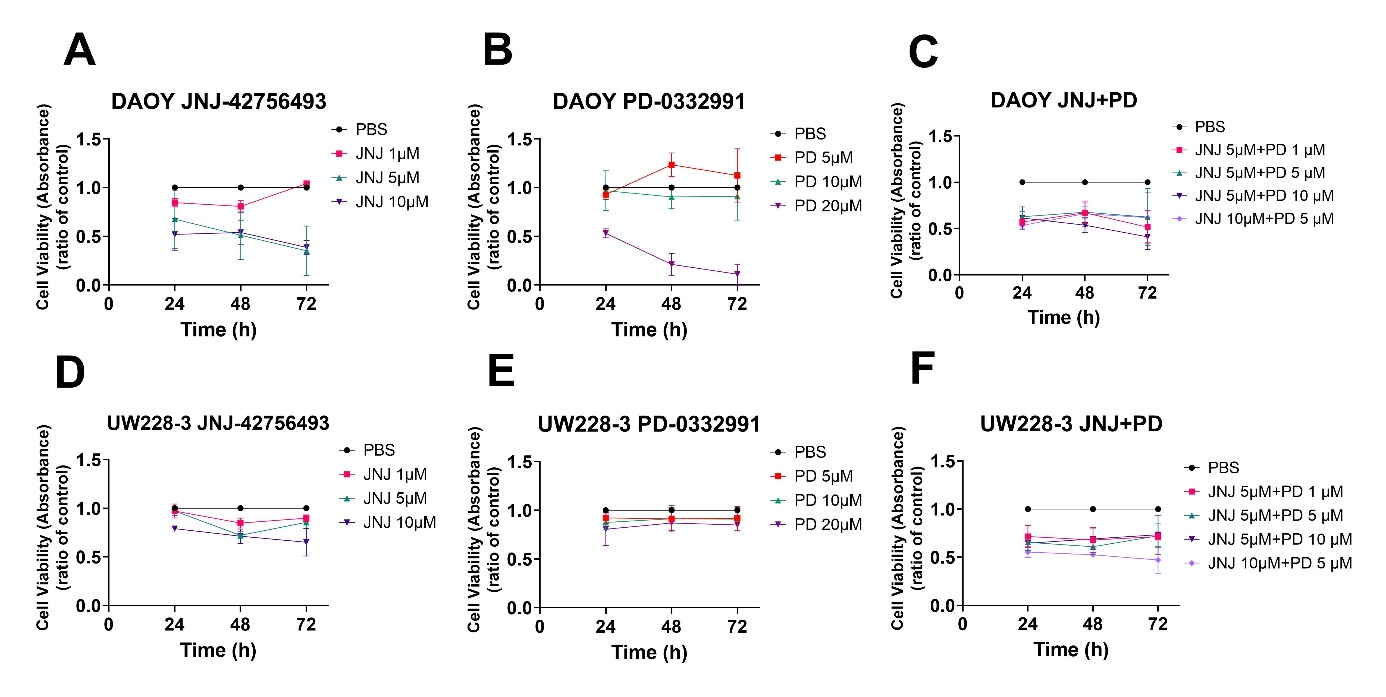


**Supplementary Figure 1: WST-1 viability assays after single and combinational treatment of FGFR and CDK4/6 inhibitors on two MB cell lines.** Viability analysis measured as absorbance, after treatment for 24, 48 and 72 h with FGFR inhibitor, JNJ-42756493 (A and D); and CDK4/6 inhibitor, PD-0332991 (B and E), on DAOY, and UW228-3 respectively. Their combinations are shown in C for DAOY and F for UW228-3. The graphs represent mean values ± standard deviation (SD) from three experimental runs per cell line. JNJ: JNJ-42756493 and PD: PD-0332991.


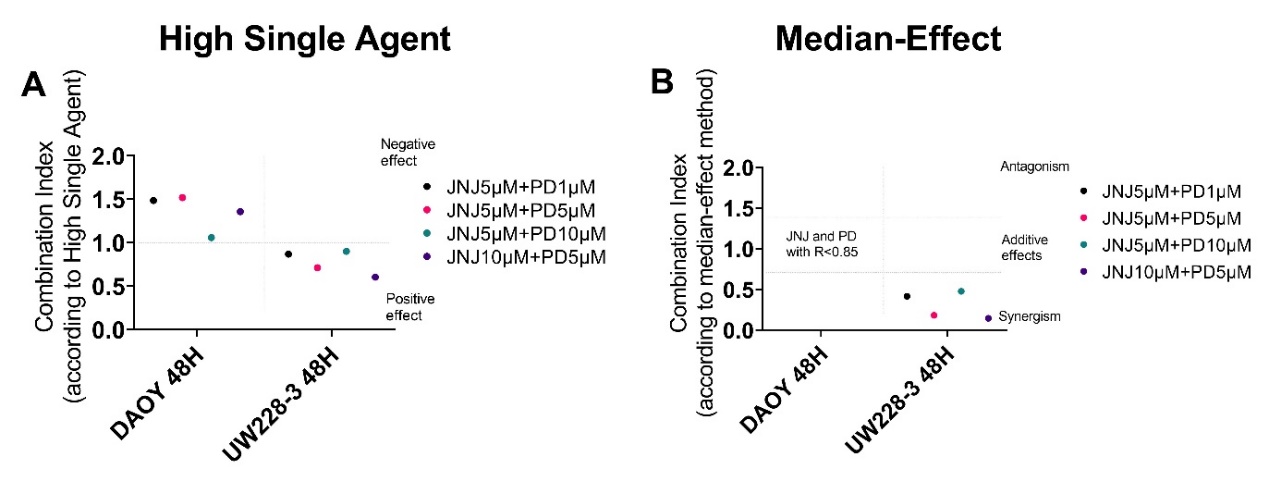


**Supplementary Figure 2:** **Combination treatments with JNJ-42756493 and PD-0332991 in MB cell lines. Combination index (CI) analyses were calculated in MB cell lines treated for 48 h by using A) the Highest Activity Agent method and B) the median-effect method.** In A chart, CI<1 indicates positive combination effect and CI>1 negative effect, while in B CI<0.7 suggests synergy, CI>1.45 antagonism, and 0.7<CI>1.45 additive combinational effects. CIs were calculated from the means of three experiments, analysed with WST-1.


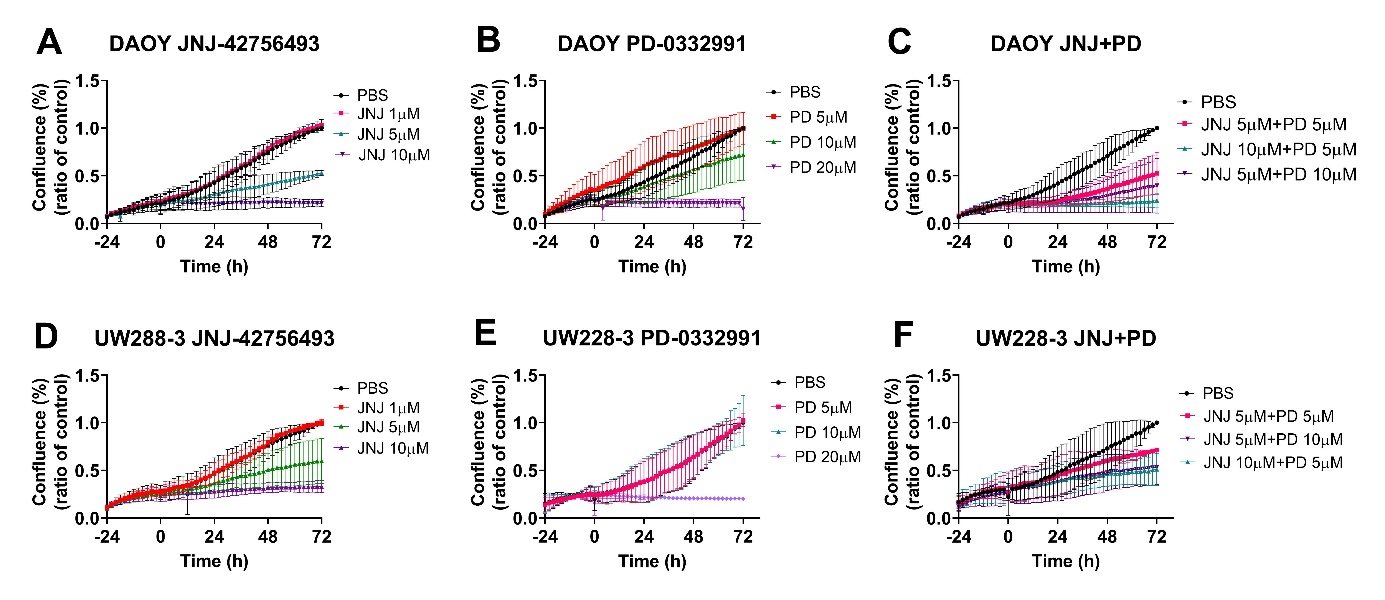


**Supplementary Figure 3:** **Cell confluence after single and combination treatment with FGFR (JNJ-42756493), and CDK4/6 (PD-332991) inhibitor on cell lines DAOY, and UW228-3.** Cell confluence was measured with the Incucyte S3 Live® Cell Analysis System. Single treatments are shown for JNJ-42756493 in A, D; and for PD-332991 in B, E. Combination treatments for JNJ-42756493 and PD-0332991 are shown in C, F. JNJ: JNJ-42756493 and PD: PD-0332991.


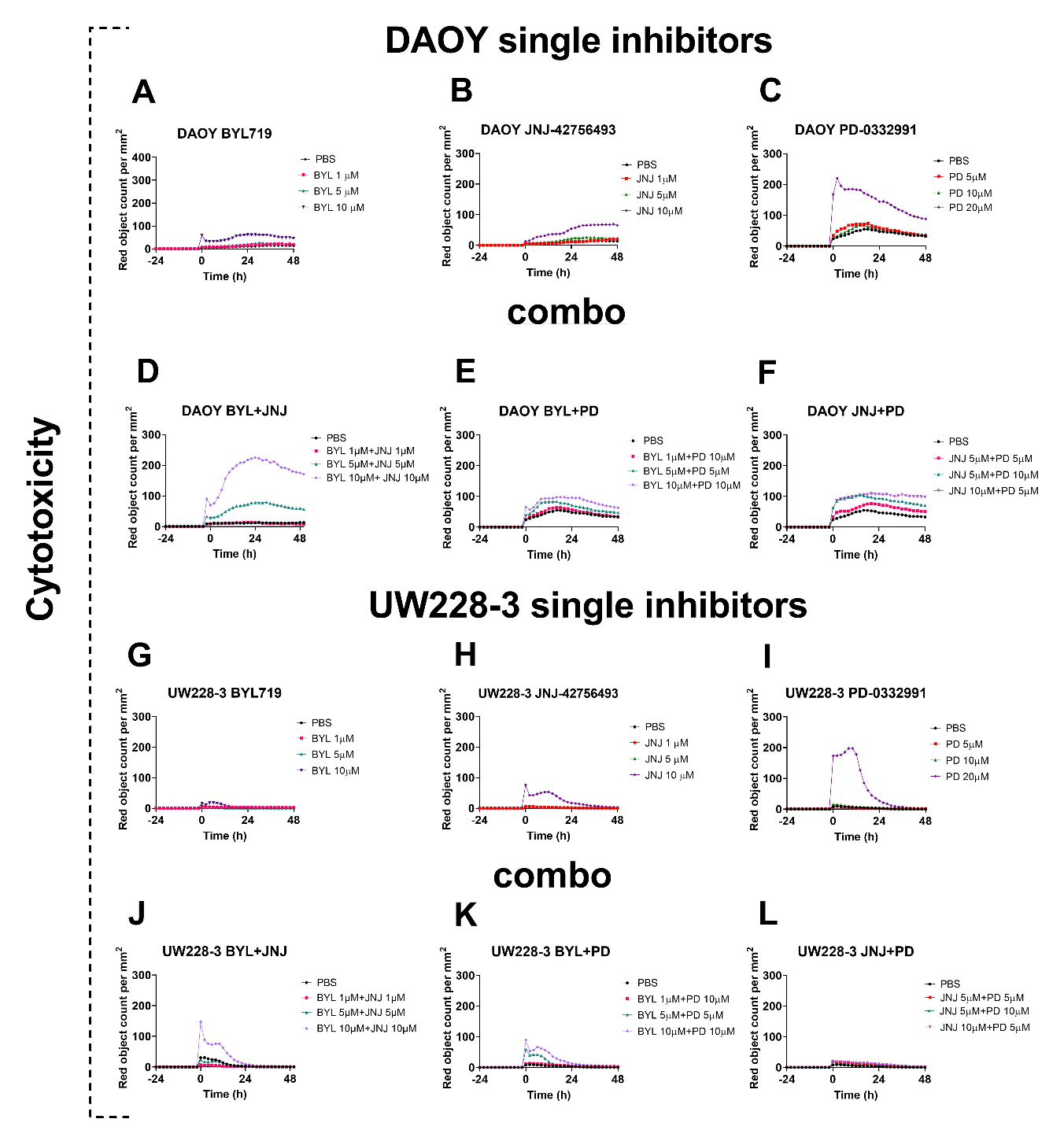


**Supplementary Figure 4. Analysis of cytotoxicity of the single and combination treatments with BYL719, JNJ-42756493 and PD-0332991, using the IncuCyte Red Cytotoxicity Assay on DAOY, and UW228-3.** 24 h after seeding, DAOY and UW228-3 respectively were treated with BYL719 (A and G); with JNJ-42756493 (B and H); and PD-0332991 (C and I) respectively, and followed up to 48 h (72 h after seeding). Corresponding combinations are shown for DAOY (D-F) and UW228-3 (J-L). The graphs represent one of three experiments.


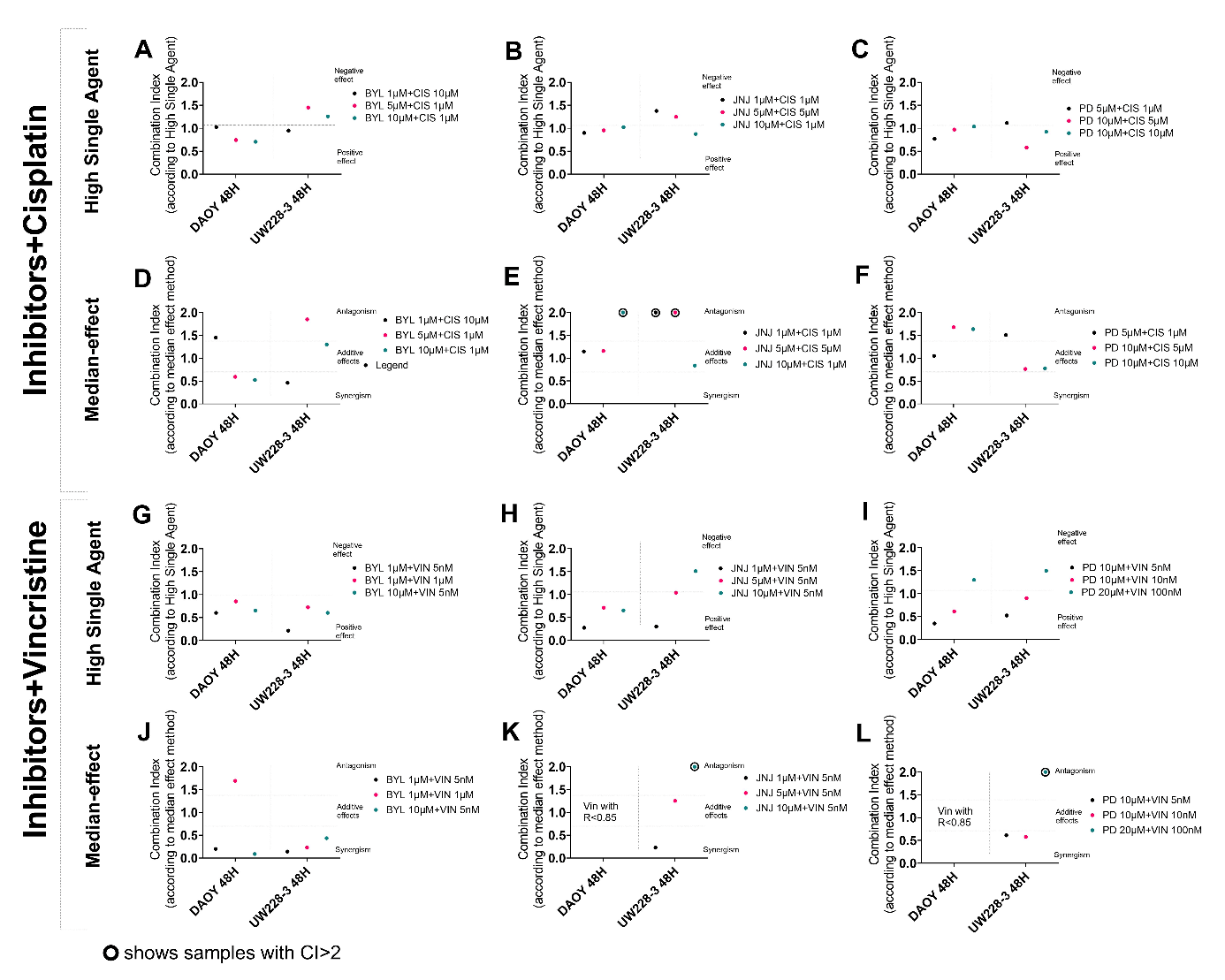


**Supplementary Figure 5. Combination treatments with BYL719, JNJ-42756493 and PD-0332991 and cisplatin or vincristine in MB cell lines DAOY and UW228-3.** Combination indexes (CI) were calculated in MB cell lines treated for 48 h by using the Highest Activity Agent method (A: BYL-CIS; B: JNJ-CIS; C: PD-CIS; G: BYL-VIN; H: JNJ-VIN; I: PD-VIN) and the median-effect method (D: BYL-CIS; E: JNJ-CIS; F: PD-CIS; J: BYL-VIN; K: JNJ-VIN; L: PD-VIN). CIs were calculated from the means of three experiments, analyzed with WST-1. BYL: BYL719, JNJ: JNJ-42756493, PD: PD-0332991, CIS: cisplatin, VIN: vincristine.


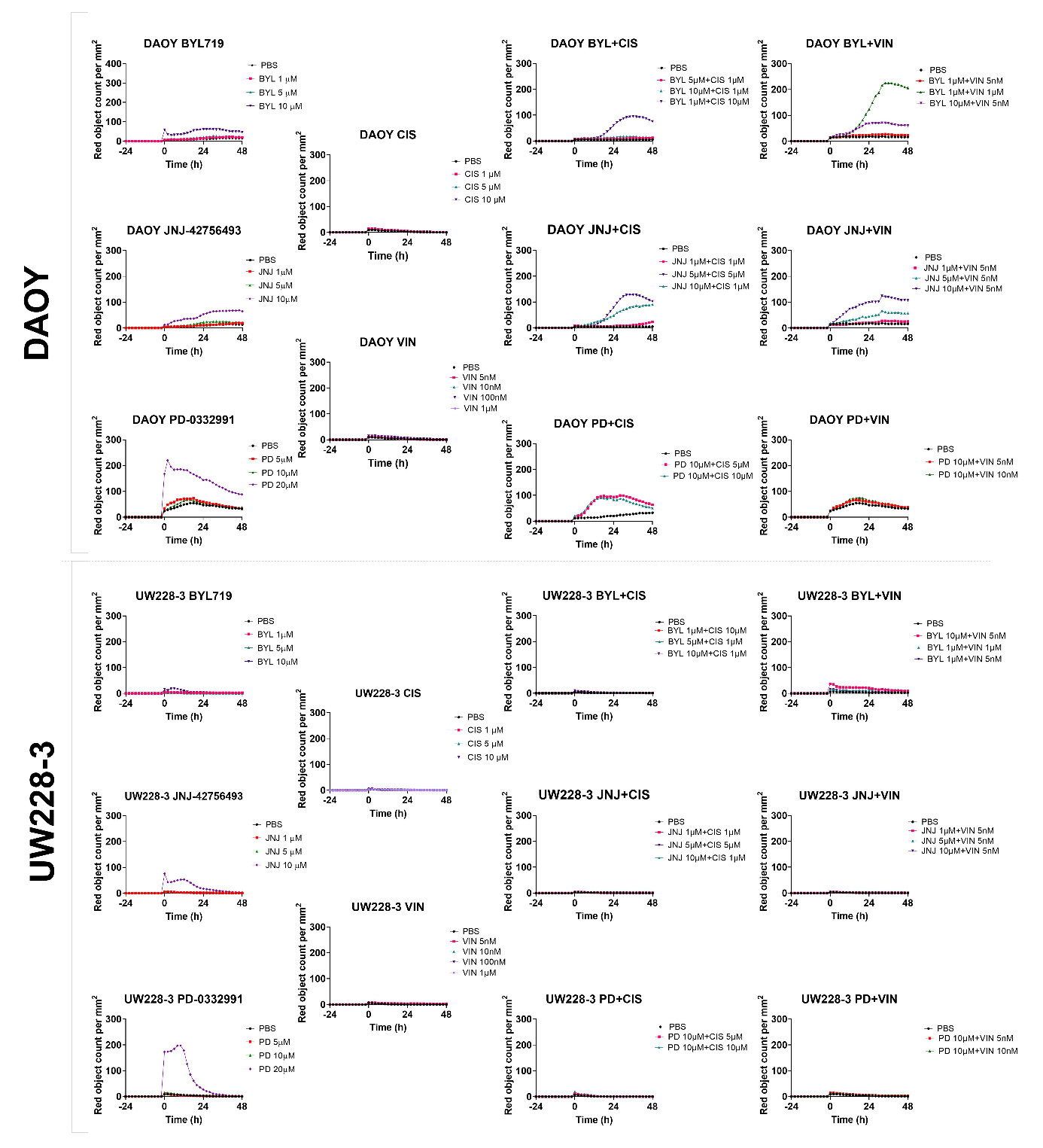
**Supplementary Figure 6. Cytotoxic effects after single treatments with BYL719, JNJ-42756493 and PD-0332991, cisplatin, vincristine and their combinations using the IncuCyte Red Cytotoxicity Assay on the MB cell lines DAOY and UW228-3.** 24 h after seeding, cells were treated with the drugs and the assays were performed up to 48 h after treatment (0 is the treatment starting point) with BYL719 (A L); JNJ-42756493 (B, M); PD-0332991 (C, N); cisplatin (D, O) and vincristine (E, P) on DAOY and UW228-3 respectively. Their combinations are shown for DAOY (F-K) and UW228-3 (Q-V). The graphs represent one out of three typical experiments. BYL: BYL719, JNJ: JNJ-42756493, PD: PD-0332991, CIS: cisplatin and VIN: vincristine.


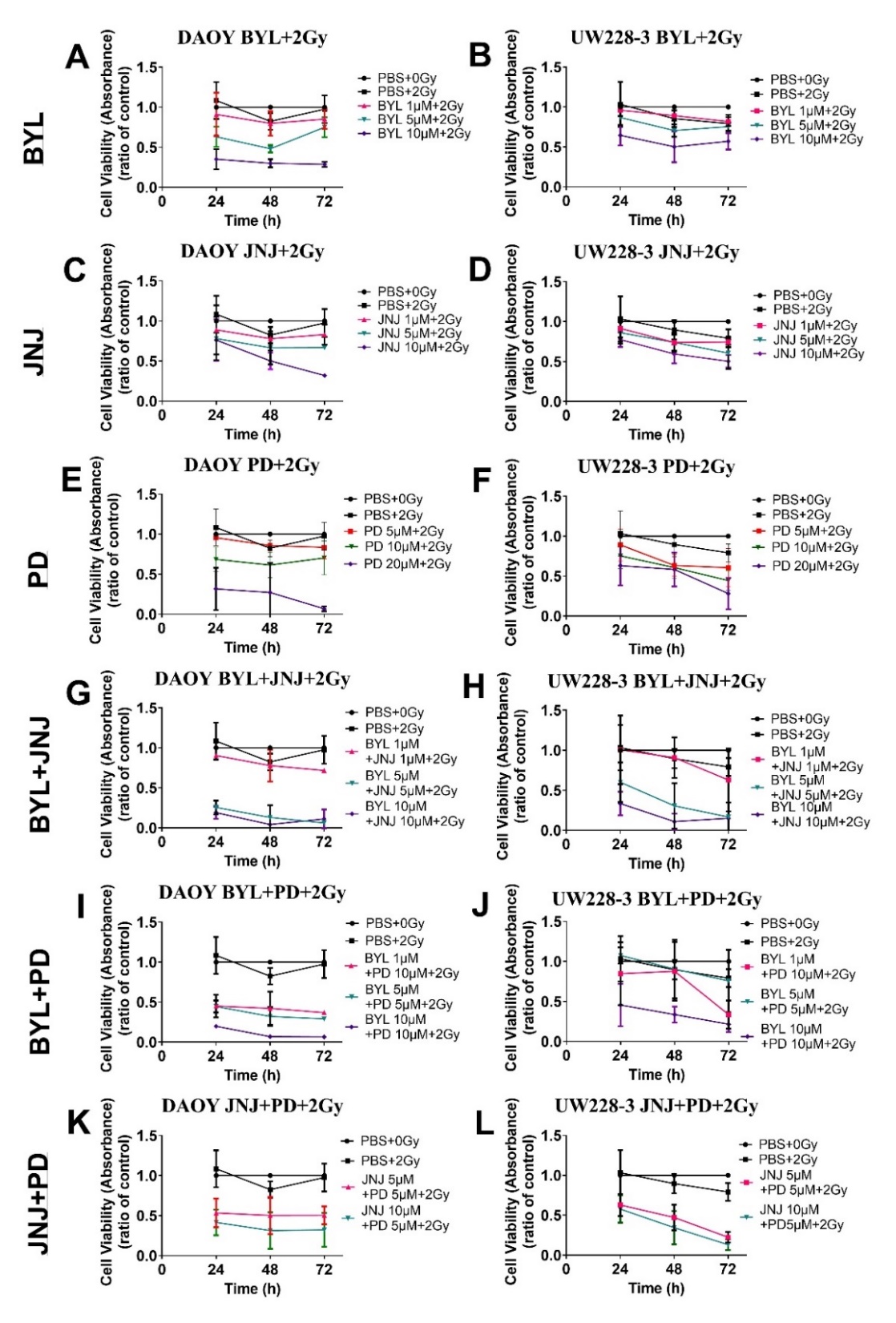


**Supplementary Figure 7:** **WST-1 viability assays after 3h pre-treatment of the 2 MB cell lines with single and combinational treatment of PI3K, FGFR and CDK4/6 inhibitors with 2 Gy irradiation.** Viability analysis measured as absorbance, after treatment for 24, 48 and 72 h with PI3K inhibitor, BYL719 (A and B) FGFR inhibitor, JNJ-42756493 (C and D); and CDK4/6 inhibitor, PD-0332991 (E and F), on DAOY, and UW228-3 respectively. Their combinations are shown for DAOY (G, H, I) and for UW228-3 (H, J, L). BYL: BYL719; JNJ: JNJ-42756493 and PD: PD-0332991, Gy: Gray.
